# Challenges and chicken production status of poultry producers in Bishoftu, Ethiopia

**DOI:** 10.3382/ps/pez343

**Published:** 2019-07-02

**Authors:** Yared A Ebsa, S Harpal, Gebeyehu G Negia

**Affiliations:** 1 Department of Animal Production and Technology, Woldia University, Mersa, Ethiopia; 2 College of Veterinary Medicine and Agriculture, Addis Ababa University, Bishoftu, Ethiopia

**Keywords:** chicken, challenges, production, Bishoftu, Ethiopia

## Abstract

The study was conducted to evaluate the current status and constraints of chicken production among poultry producers in the study area. A high percentage of poultry producers were male (69.2%) and married (53.8%). More than half had at least a tertiary education (53.8%) followed by those with secondary schooling and a primary education. The average age of poultry producers was 33.2 ± 11.0 years and this ranged from 16 up to 65 yr of age. Among all the producers’ samples, 36.5% reared chicks while hens were the largest flock type followed by pullets, chicks, cockerels, and cocks. A large proportion of producers (94.2%) provided supplementary feed to their birds and most (82.7%) of them utilized commercial ration. Mostly, 38.5% of poultry producers provide supplementary feed any time depending upon the amount of feed left in the feeding trough. The 3 biggest challenges to producers were sudden outbreak of disease, the cost of commercial feed, and the lack of availability of day-old chicks.

## INTRODUCTION

Ethiopia is endowed with many livestock species with an estimated population of 56.7 million cattle, 29.33 million sheep, 29.11 million goats, and 56.87 million poultry (CSA, 2015). Poultry production stimulates local economic development of urban centers through the development of related micro-enterprises wholly or partly responsible for the provision of inputs and processing, packaging, and marketing of outputs as well as the provision of services to the sector (Mutami, 2015). In addition, it may contribute to poverty alleviation and socio-economic inclusion of vulnerable groups such as the urban poor, women, the disabled, orphans, and the unemployed to provide them with a decent livelihood (Gororo and Mabel, 2016). Poultry in Ethiopia provides the following benefits: production of eggs for hatching, sale and home consumption, and production of birds for sale, processing, replacement, and home consumption (Tadelle et al., 2007). Chicken have a short generation interval and higher feed conversion efficiency, thus providing a cheap source of animal protein and also chicken meat is the most palatable and easily digestible animal meat and contains essential amino acids required for human beings, and eggs are richly endowed with nutrients (Lahkotia, 2002). Chickens also have a socio-cultural and religious role mainly in the rural communities throughout Ethiopia (ESAP, 2009). The productivity of poultry has been limited by the scarcity and subsequent high prices of conventional protein and energy sources in Ethiopia. Poultry producers in Ethiopia are constantly complaining about the high cost and quality of poultry feed on the market. The quality of mixed feed used is generally poor and most formulations available do not have vitamin or mineral premixes (EIAR, 2016). The price of raw materials varies according to the source of supply and region. Prices of mixed feed remain excessively high even at times when the price of the major component of mixed rations fall by more than 50% (Tadelle and Ogle, 2001). Thus, the use of cheap and readily available local feed resources has the potential to increase poultry productivity (Lukuyu et al., 2011). Therefore, the present survey was conducted to assess the challenges and chicken production status of poultry producers in Bishoftu.

## MATERIALS AND METHODS

### Description of the Study Areas

The study was conducted in Bishoftu, Ethiopia. Bishoftu is in the central highlands of Ethiopia, 8°45′ North latitude and 38°59′ East longitude. The town is located in tepid to cool sub-moist mid-highland at an altitude of about 1,920 m a.s.l. with moderate weather conditions. It is located about 45 km southeast of Addis Ababa. The area has an altitude of about 1,900 m a.s.l., with an average annual rainfall of 686.9 mm. The average minimum and maximum temperature range from 10.9 to 27.0°C with a mean value of 18.9°C. The average relative humidity is 60.0% (DZARC, 2001).

### Methods of Data Collection and Analysis

Both qualitative and quantitative data were collected using standard pre-tested semi-structured open- and closed-ended questionnaires based on the flock ownership status of poultry producers. The qualitative data included sex, marital, and educational status; and flock size of poultry producers. Other qualitative data were poultry ownership status, feeding and feed resource utilization, and potential constraints of chicken production. On the other hand, the quantitative data were age, family size, and flock size. Focus group discussions were carried out in order to investigate recommendations based on poultry producers’ point of view. Finally, the collected data were organized using Microsoft Excel and analyzed using statistical package for social sciences (SPSS, 2015). Chicken production constraints were ranked using the formula adopted from Kosgey (2004) and the variable with the highest index value is the highest economically important.

$$\begin{equation*}
\rm{Index}=\frac{\sum[(n\times \rm{no\,\, of\,\, p\,\,1st}) + \rm{(n-1\times no\,\, of\,\, p\,\, 2nd)} + \ldots + \rm{(1 \times no\,\, of\,\, p\,\, last)}]}{\sum[\rm{(n\times no\,\, of\,\, p \,\, 1st)} + {{\rm (n}-1\times no\,\, of\,\, p\,\, 2nd)} + \ldots + \rm{(1\times no\,\, of\,\, p \,\,last)}] \rm{for\,\, all\,\, factors}},\end{equation*}$$

where p = problem n = value given for the least ranked factor; no = number.

## RESULTS AND DISCUSSION

### Socioeconomic Characteristics

The socioeconomic characteristics of the poultry producers are presented in Table 1. The study indicated that almost half of poultry producers (48.9%) in Bishoftu were located in Babogaya whereas 17.0% and 10.6% of them were located in Dembi and Chelekleka, respectively. Larger percentages of poultry producers (69.2%) in Bishoftu were males. Similarly, larger percentages of poultry producers (82.0%) in Bangui (Keita, 1999) were males, respectively. Larger percentages of poultry producers (53.8%) in Bishoftu were married, which agreed with the findings of Adedeji et al. (2014). More than half of poultry producers (53.8%) had a tertiary education followed by those who attended secondary and primary education, which did not agree with the observations of Nebiyu (2016). The average age of poultry producers of the present study was 33.2 ± 11.0, ranging from 16 up to 65 yr. On the other hand, Adebayo and Adeola (2005) and Bamiro et al. (2013), had reported ages ranging from 20 to 40 and 31 to 50 yr, respectively.

**Table 1.** Socio-economic characteristics of poultry producers in Bishoftu.

Parameters	Babogaya	Ayerhail	Chelekleka	Dembi	Gebeya	Kajima	Kurkura	Total
Poultry producers	48.9	3.2	10.6	17.0	9.6	6.4	4.3	100.0
Sex								
Male	28.9	2.5	5.1	16.3	7.5	3.8	5.1	69.2
Female	18.2	0.8	4.7	2.4	2.4	2.4	0.0	30.8
Marriage								
Single	22.0	1.2	4.6	12.7	1.2	2.3	2.3	46.2
Married	23.1	1.7	5.1	4.3	6.8	3.4	1.7	53.8
Education level								
Illiterate	6.5	0.0	0.4	1.9	0.8	0.0	0.0	9.6
Primary education	7.3	0.0	0.5	2.9	2.2	0.0	0.5	13.5
Secondary education	7.0	0.0	4.0	4.0	2.1	4.0	0.0	23.1
Tertiary education	16.6	4.1	8.3	12.4	0.0	6.2	6.2	53.8

### Flock Structure and Size

The flock structure of poultry producers is presented in Figure 2. Approximately one-third (36.5%) of

poultry producers kept chicks. The result was in agreement with the findings of Wondu et al. (2013) who stated that in Northern Gonder (Ethiopia) flocks were dominated by chicks (47.0%), hen (20.2%), cocks (9.5%), pullets (14.8%), and cockerels (8.5%).

**Figure 1.**
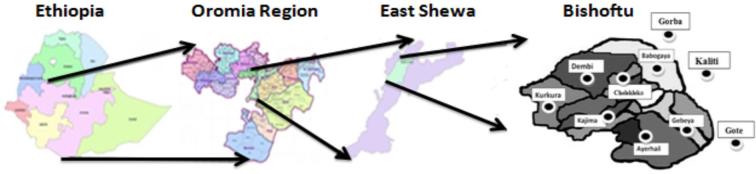
Geographical location of the study areas.

**Figure 2.**
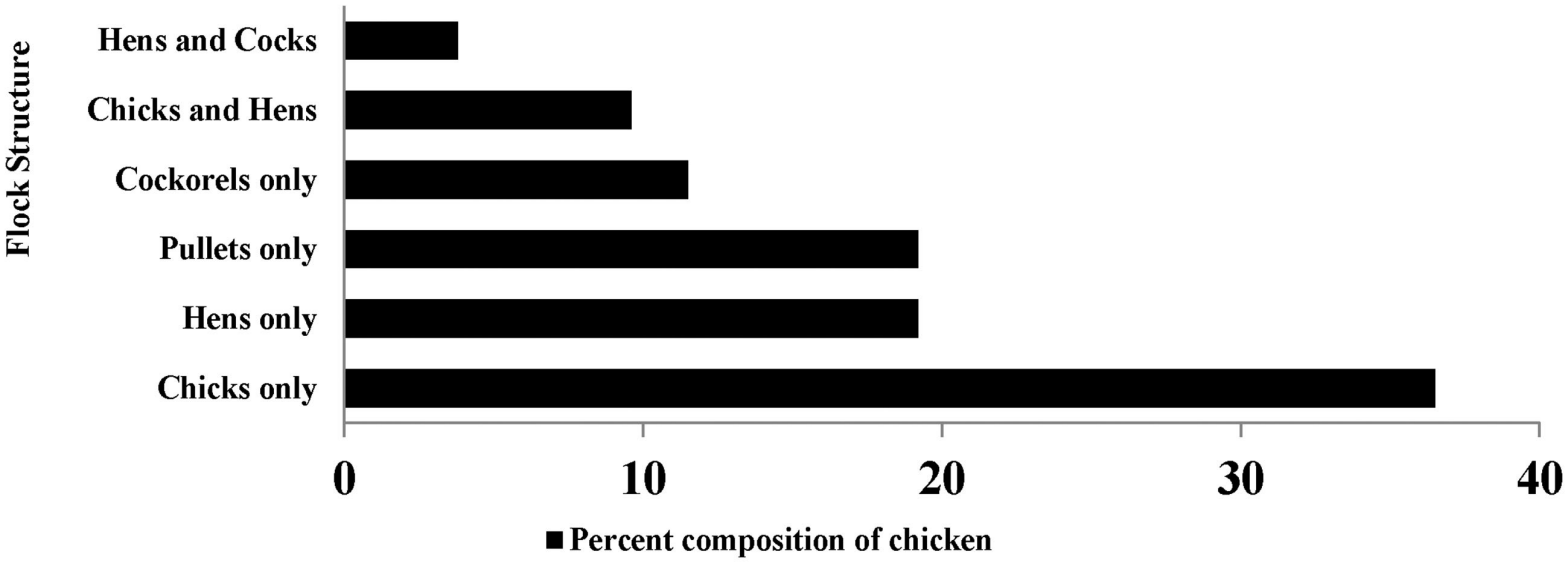
Flock structure of poultry producers.

The average flock size of poultry producers is presented in Table 2. The highest average flock size was represented by hens (2380.9) followed by pullets, chicks, cockerels, and cocks, respectively. The minimum and maximum flock size recorded under intensive poultry production system in Ethiopia were approximately 2,500 and 50, 000 specialized breeds (FAO, 2007).

**Table 2.** Average flock size of poultry producers in Bishoftu.

Types of flock	Chicks	Cockerels	Pullets	Hens	Cocks
Flock size (μ ± SD)	1293.3 ± 823	1,160 ± 448.1	2133.7 ± 3200.3	2380.9 ± 2980.3	4 ± 1.4
Range	10 to 3,000	700 to 2,000	50 to 11,000	6 to 10,000	3 to 5

### Feeding and Feed Resource Utilization

Housing, feeding, and feed resource utilization among poultry producers is presented in Table 3. Almost all (98.0%) of poultry producers used a house confined with deep litter. Larger percentages (94.2%) of poultry producers provided supplementary feed to their chicken. A higher percentage (82.7%) of poultry producers fed a commercial ration while 17.3% fed mixed feed to their chicken. Similarly, a study conducted by Tadesse et al. (2017) stated that larger percentages (61.5%) of intensive chicken farms in Tigray (Ethiopia) used commercial feed as a major source of feed. In the present findings, many poultry producers (38.5%) fed their chicken any time of the day depending on the amount of feed left on the feeding trough, 36.5% fed their chicken 3 times per day, 25.0% fed their chicken 2 times per day. This is different than what was reported by Tadesse et al. (2017) who found that 69.2% of poultry producers fed their chickens 3 times per day while a lower percentage fed their chickens once (9.6%) and twice (21.2%) per day, respectively.

**Table 3.** Feeding and feeding frequency of poultry producers in Bishoftu.

Parameters	% of Poultry producers
Scavenging and supplement	5.8
Supplement only	94.2
Supplemental feeds	
Commercial rations	82.7
Mixed feed	17.3
Feeding frequency	
Twice per days	25.0
Three times per day	36.5
Any time	38.5

### Major Constraints of Poultry Producers

The major constraints of poultry producers are presented in Table 4. The 5 major constraints of chicken production in the present study were sudden disease outbreak (1st), the high cost of commercial ration (2nd), unavailability of day-old-chicks in time (3rd), market instability and poor sales (4th), and poor supply and quality of vaccine (5th). In Addis Ababa (Ethiopia) the high price of feed, shortage of land, unavailability of pullets in time, high cost of pullets, feed quality, shortage of water, lack of available feed in nearby areas, marketing difficulties during selling of poultry products, health problem, lack of access to credit, and inadequate training were reported by Nebiyu (2016). On the other hand, major constraints of chicken production among poultry producers under an intensive system in Tigray (Ethiopia) collectively were lack of knowledge to prepare mixed feed, the high price of mixed feed, unavailability of commercial feed in nearby area and unavailability and cost of feed ingredients (Tadesse et al., 2017).

**Table 4.** Major constraints of poultry producers.

Constraints	1st	2nd	3rd	4th	5th	6th	Index	Rank
Disease outbreak	72	21	9	3	3	0	0.180	1
High cost of commercial ration	30	48	12	12	12	6	0.164	2
Unavailability of day old chicks in time	15	9	39	9	3	0	0.099	3
Market instability and poor sales	12	9	12	24	18	6	0.085	4
Poor supply and quality of vaccine	3	3	9	21	33	27	0.069	5
Cold season	6	18	6	18	3	0	0.064	6
Unavailability of pullets in time	0	0	30	15	9	3	0.057	7
High cost of labor and management	0	3	9	15	27	15	0.051	8
Poor quality of commercial ration	6	12	3	12	3	3	0.047	9
High cost of rental house	6	12	6	9	0	3	0.046	10
